# Nutritional Education in Polish Companies: Employee Needs and the Role of Employers in Health Promotion

**DOI:** 10.3390/nu16193376

**Published:** 2024-10-04

**Authors:** Anna Katarzyna Mazurek-Kusiak, Andrzej Soroka, Agnieszka Godlewska

**Affiliations:** 1 Department Tourism and Recreation, University of Life Sciences in Lublin, 15 Akademicka Street, 20-950 Lublin, Poland; 2 Faculty of Medical and Health Sciences, University of Siedlce, Stanisława Konarskiego 2, 08-110 Siedlce, Poland; andrzej.soroka@uws.edu.pl (A.S.); agnieszka.godlewska@uws.edu.pl (A.G.)

**Keywords:** healthy eating, employee, employer, company canteens, barriers and motives

## Abstract

The modern work environment is constantly evolving, and with it, the emphasis on employee health and well-being is increasing. Background: Nutritional education has become a key component of health promotion strategies in many companies that recognize the benefits of healthy eating habits for enhancing efficiency and job satisfaction. Objectives: The aim of this study was to understand employees’ nutritional needs at work, and to assess the support provided by employers. The analysis focused on employees’ eating habits and their attitudes towards eating at work, motives and barriers to healthy eating. Methods: The study was conducted using an anonymous survey completed by 1.056 individuals from across Poland. A discriminant function was selected for data analysis, which examined the differences between groups. Results/Conclusions: The larger enterprises are more inclined to establish meal consumption spaces, which contributes to a more organised work culture. The larger the business, the more time employees spend eating meals, it being influenced by better-developed eating facilities, which undoubtedly encourages the workers to take longer breaks. The people with obesity were found to place the highest value on healthy food options and the people with a normal BMI are more responsive to healthy food subsidies, nutritional advice provided by a dietitian, and access to fitness facilities.

## 1. Introduction

Modern society encounters numerous health challenges, the majority of which stem largely from poor dietary habits such as: a decrease in the intake of fresh or minimally processed meals and an increase in the consumption of ultra-processed goods. It was brought on by changes in lifestyle during the previous several decades. A bad diet and other health issues, such as being overweight, are signs of new eating habits [1]. According to estimates, the total yearly costs of non-adherence to healthy lifestyles in the United States are in the hundreds of billions of dollars [2]. This is a global problem but, through properly conducted nutritional education, it can lead to an increase in people’s awareness of the importance of a healthy lifestyle [3]. Companies play a distinctly important role in this process as they can influence their employees’ health by promoting healthy habits while also providing appropriate working conditions [4].

In recent years, there has been growing interest in the impact of nutritional education on employee health [5] and the role of employers in promoting a healthy lifestyle among their staff [6,7,8]. Numerous studies underscore the significant benefits of implementing health programs in the workplace. These programs contribute to the improvement of employees’ physical and mental health, boost their productivity and increase their engagement in maintaining a healthy lifestyle [9,10,11].

Nutritional education can significantly contribute to improving employees’ dietary habits, which in turn can lead to a reduced risk of diet-related diseases such as obesity, type 2 diabetes, and cardiovascular diseases [7]. Nutritional education in the workplace encompasses an array of activities, including workshops, seminars, one-on-one counselling sessions with a nutritionist, and informational campaigns [12].

Employers should play a key role in promoting health among employees by creating a supportive work environment and implementing programs which encourage a healthy lifestyle [13] which can significantly affect the well-being of their employees [14]. These programs frequently include initiatives such as providing healthy meals in canteens, organising health-promoting days, and offering Wellness packages which may include fitness club memberships or access to dietary counselling [15].

Implementation of health programs in the workplace carries numerous benefits for both employees and employers [16]. Research has shown a decline in sickness absences, boosted productivity and improved employee well-being. In economic terms, investing in employees’ health can lead to substantial savings related to healthcare costs and a reduction in the number of sick leave days [17].

Despite the numerous benefits of implementing health programs, companies encounter a number of challenges. The most commonly cited issues include budget constraints, a lack of employees’ awareness about the benefits of such programs, and difficulties in engaging employees [18]. A key element of success is the proper planning and promotion of these initiatives [19] to effectively reach as many employees as possible.

In Poland, the topic of nutritional education in the workplace is a relatively new issue which is gaining importance in the context of the increasing number of employees suffering from diet-related diseases [5]. Understanding the nutritional needs of employees and the role of employers in promoting a healthy lifestyle are crucial for the effective implementation of health-related programs in companies.

The present paper aims to examine the current needs of Polish employees rerated to healthy nutrition, and assess the extent to which employers engage in promoting health in the workplace. The aim is also to show under what conditions Polish employees eat their meals (place, time devoted to lunches), what are the factors motivating employees to eat healthily in the workplace and what are the barriers in this respect.

The following research hypothesis is set in the study.

Polish employees often eat at their workplace in a hurry. The main barrier limiting healthy eating is lack of time and financial resources, so the motivation for a healthy lifestyle would be an in-company canteen where dietary, healthy and nutritious meals can be purchased, access to free advice from a dietician and the possibility to use the fitness room during working hours.

## 2. Materials and Methods

### 2.1. Participant

The research relied on a diagnostic survey method with a direct questionnaire as the technique. The authors developed a two-part questionnaire whose first part included questions about various topics, such as the location and timing of meal consumption, employer support for healthy nutrition, motivating factors for healthy eating in the workplace, and barriers encountered in implementing these tasks. The second part contained questions to determine the characteristics of the respondents, including gender, age, the size of the company they work for, height and weight which were used to calculate the Body Mass Index (BMI). The study conformed to the code of ethics of the World Medical Association and the standards for research recommendations of the Helsinki Declaration. The study was anonymous. The protocol was approved by the local university ethics committee at the Siedlce University of Natural Sciences and Humanities no. 1/2018 from 16 November 2018.

The study was conducted between 2022 and 2023 across Poland, involving 1056 employees from Polish companies. The criteria for selecting a representative sample were based on proportional sampling, considering gender and company size, until the calculated quotas were met (see Table 1).

A micro- or small enterprise is defined as a company employing up to 49 employees and with an annual turnover or total balance sheet not exceeding 10 million euros. A medium-sized enterprise is a company with 50 to 249 employees and with an annual turnover of up to 50 million euros or a total annual balance sheet not exceeding 43 million euros. A large enterprise employs at least 250 people or has a turnover exceeding 50 million euros or a total annual balance sheet greater than 43 million euros [20].

### 2.2. Statistical Analysis

Statistical calculations were performed using Statistica 13.1 PL software. Relationships between nominal and ordinal variables were examined utilising pivot tables which synthetically presented the emerging associations using the χ^2^ statistic, Pearson’s contingency coefficient C, and Cramer’s V statistic. The χ^2^ statistic was calculated using Formula (1):

(1) $$\chi^{2}=\overset{k}{\underset{j=1}{\sum }}\frac{{\left(O_{j}-E_{j}\right)}^{2}}{E_{j}}$$

where:

χ^2^—chi-square test,
*Oj*—observed frequency for a given group,
*Ej*—expected frequency for a given group.

The discriminant function was also used for data analysis, it examining differences between groups based on a set of selected independent variables, using Formula (2):

(2) $$D_{\mathrm{kj}}=\beta_{0}+\beta_{1}x_{1\mathrm{kj}}+\cdots +\beta_{p}x_{\mathrm{pkj}}$$

where:

p—number of discriminant variables,
β—discriminant function coefficient,
D_kj_—canonical value of the discriminant function for the k-th case in the j-th group,
K = 1, …, n,
j = 1, …, g,
x_kj_—value of the i-th discriminant variable for the k-th case in the j-th group.

The main objective of using discriminant analysis was to predict case classifications. In the study, classification functions employed were determined for each segment of employees and enterprises. Each case was classified into the group with the highest classification value. Differences and relationships were considered statistically significant at the level of *p* < 0.05.

## 3. Results

In the first stage of the research, respondents were asked about the support employees receive from their employees regarding healthy nutrition.

The discriminant function model included four out of six types of support provided by employers to promote nutritional education. The highest values the classification function achieved were found for meal breaks which allowed employees to restore their energy and motivation to continue work. This support was most commonly implemented in large enterprises where it reached a classification function value of 4.777 followed by medium-sized enterprises with a value of 4.169. Training sessions on proper nutrition, which were also most frequently offered by large enterprises, achieved a classification function value of 2.721. Another type of support was that of a nutritionist who was employed to develop tailored diet plans for specific employees. This form of support was also predominant in large companies, with a classification function value of 2.018. The lowest classification function values were observed in each of the studied groups for support in the form of complementary food such as fruits, vegetables, or juices. This type of support was most commonly found in medium-sized enterprises. Canteens for employees, available kitchen facilities and equipment for meal preparation were excluded from the model, as these forms of support are relatively uncommon in Polish companies (Table 2).

In the second stage of the research, employees were asked about the location and time allocated for meals at their workplaces.

A statistical analysis conducted using the chi-square test revealed a significant difference (chi-square test = 117.316, *p* < 0.001) in meal consumption places depending on the size of the company. The Cramer’s V coefficient value of 0.236 indicates a moderate strength of the relationship between the variables (Table 3).

In micro- and small enterprises, 32.71% of employees reported eating meals directly at their workstations during breaks, and 31.12% at their workstations while performing job duties. In medium-sized enterprises, 30.28% of employees consumed meals in designated spaces, and 45.11% at their workstations during breaks. In large companies, 28.37% of employees ate meals in designated spaces, 52.62% at their workstations during breaks, 11.02% at their workstations while working, and 3.31% at other places.

The time allocated for meals in workplaces across Poland was analysed in terms of the size of the organisation. The Cramer’s V coefficient of 0.318 indicates a moderate strength of an association between the company’s size and the time allocated for meal consumption. In micro- and small enterprises, the largest percentage of employees, 28.72%, spent up to 5 min on meal consumption, while 13.83% reported spending more than 30 min. In medium-sized enterprises, 11.67% of respondents spent up to 5 min eating meals, 44.48% spent 6–15 min, 18.93% spent 16–30 min, and 17.03% spent more than 30 min. In large enterprises, the results were as follows: 23.42%, 26.17%, 30.85%, and 14.88%, respectively (Table 4).

The third stage of the study comprised analysis of factors motivating employees to eat healthily in the workplace, and the associated barriers.

The discriminant analysis presented in Table 5 pertains to the factors motivating employees to eat healthily in the workplace, considering different BMI groups: individuals with normal weight and persons who are either overweight or living with obesity. The Wilks’ lambda value for the entire model was 0.154, indicating that the selected factors effectively differentiate between the groups of employees.

An availability of healthy food options in the canteen/catering was particularly motivating for overweight and employees with obesity, as indicated by the high values of the classification functions (5.950 and 6.277, respectively). This suggests that access to healthy food at the workplace is crucial for these groups, more so than for individuals with a normal BMI whose classification function value was 3.025. Subsidised healthy food was the most significant for employees with a normal weight (3.913), it being less motivating for overweight and persons with obesity (respectively, 2.241 and 2.771). Nutritionist counselling paid for by the company were more motivating for individuals with normal weight (3.609) compared to those being overweight (1.917) and with obesity (2.399). Access to a fitness room was more motivating for those with a normal BMI value (3.165) than for those who were overweight (2.398) and with obesity 1.875). Stocking vending machines/on-site shops with healthy snacks instead of sweets and salty snacks was particularly important for overweight and with obesity individuals (respectively, 3.613 and 4.903). For those with a normal BMI value, this factor was less significant, with a classification function value of 2.246. The values of the constants in the classification function, which were 27.017 for individuals with a normal BMI, 27.001 for overweight employees, and 34.573 for people with obesity, indicate differences in the baseline motivation levels among these groups (Table 5).

Discriminant analysis in terms of barriers to healthy nutrition in the workplace was also conducted considering employees’ body mass index (BMI). The Wilks’ lambda value of 0.136 indicates that the selected barriers effectively differentiate between employee groups based on their BMI (Table 6).

The most significant barrier for individuals who were overweight and with obesity was budget constraints, which received the highest classification function values of 5.700 and 5.954, respectively. Time constraints were the second most significant barrier, particularly for those with obesity, the classification function value being 5.121. For individuals with a normal BMI value, the main barriers included lack of knowledge (classification function value of 3.822) and lack of motivation (classification function value of 3.656). Logistic constraints, such as the availability of healthy food, were more significant for those with a normal BMI, while they were less important for individuals who were either overweight or with obesity (Table 6).

## 4. Discussion

Nutritional education in the workplace and engagement of employers in healthy lifestyle promotion have a positive impact on employees’ health, performance and productivity. While implementing health programs in Polish enterprises faces challenges, it is an investment that can yield tangible benefits for both employees and employers. In the above research on employee support for healthy eating, four main forms of support provided by employers were identified: meal breaks, free food, nutrition-related training and access to a dietician. Analysis of the results shows that meal breaks were the most important, and were clearly more common in large and medium-sized companies. When it came to the availability of healthy food options in canteens, large companies received the highest value for the classification function, suggesting that they are the ones most likely to offer this kind of support. However, Polish employers have different approaches to supporting employee nutrition education. This support is the most pronounced in large companies which offer meal breaks, allowing employees to regain physical energy and, thus, increase their work motivation. In other countries and cultural contexts, meal breaks also play a significant role in enhancing employee well-being. Research conducted in Germany highlights the positive influence of meal breaks on both physical and mental health, which translates into better performance and job satisfaction [21]. Training sessions on proper nutrition, which was also highly valued in the reported study, is another crucial element of employer support. Research by Rachmah et al. [22] has shown that nutritional education in the workplace can lead to long-term changes in employees’ eating habits, which in turn affects their health and work effectiveness. Another interesting point is employing a nutritionist who develops personalised diets for employees. As studies conducted e.g., in the United States demonstrate, such a form of support is becoming increasingly popular, particularly in industries where employees’ physical health directly affects work performance [23,24]. The least effective form of support turned out to be providing complementary food, such as fruits, vegetables or juices, which is consistent with the findings of other studies suggesting that, while such attempts are well-received by employees, their actual impact on changing eating habits and improving health is limited [25]. This can be explained by the fact that such support does not require active employee engagement, making its effects less sustainable compared to, for example, training sessions or one-to-one nutrition counselling. The lack of popularity of employee canteens and kitchens available to workers is also noticeable, which contrasts with trends observed in countries across the Americas where canteens and eating facilities are standard in many workplaces boosting employee integration and health [26].

The next part of the study focused on where and when meals were eaten. The results of the chi-square test showed significant differences in eating patterns according to the size of the establishment, with a predominance of eating at workplaces in large companies. The time spent eating also differed between companies, with a significant proportion of time spent on very short breaks in micro and small companies. In their study, Sungjin et al. [27] also noted that employees in larger workplaces tend to take longer meal breaks. Large companies were found to have better-developed eating facilities, which encourages employees to spend more time consuming their meals. These findings concur with the results of the present study which shows that in large companies the percentage of employees spending more than 15 min on meals is significantly higher than in small enterprises. The finding that work in larger companies is more varied, and roles are more specialised, allowing for more flexible time management, is confirmed by the research of Goh et al. [28]. Their study demonstrated that larger companies with more complex organisational structures often provide employees with greater autonomy in managing their time, which leads to longer meal breaks. Furthermore, Hakro et al. [29] found that a beneficial one-hour lunch break positively affects employee health and productivity, and reduces work-related stress. This type of support assists in preventing employee absenteeism, improves job satisfaction, and fosters a positive attitude towards work.

Analysis of factors motivating healthy eating showed that the availability of healthy options and subsidies for such products were particularly important for overweight and wit obesity employees. In contrast, barriers limiting healthy eating, such as budget and time constraints, were most important for those with a higher BMI. Research conducted by Geaney et al. [23] underscores the importance of healthy food availability in the workplace. According to Glympi et al. [12], the introduction of healthy food options significantly increased fruit and vegetable consumption among employees, particularly those with a higher BMI. These results agree with the present study which indicates a higher motivation among overweight and with obesity persons to take advantage of such opportunities. Additionally, the authors demonstrated that healthy food subsidies have the greatest impact on individuals with a normal body weight. Similar findings were reported by Kunzmann et al. [30] whose research demonstrated that people with a normal BMI are more likely to choose healthy food when it is financially supported. On the other hand, overweight and with obesity persons may require more direct incentives beyond subsidies, as confirmed by other researchers [12,31,32]. The study reported here also shows that advice offered by a nutritionist is more motivating for persons with normal body weight. Research by Morgan et al. [33] supports this finding, indicating that people with a normal BMI are more likely to seek and follow dietary advice, possibly due to their greater health-related awareness. Meanwhile, overweight and with obesity individuals may need a more personalised approach that addresses their specific challenges and barriers, as emphasised by Bąk-Sosnowska et al. [34]. Similar results were obtained by Maes et al. [10]. The work reported here demonstrated that access to a fitness room is more motivating for individuals with a normal BMI. Similarly, research conducted by Marin-Farrona et al. [35] showed that people with a normal body weight are more inclined to participate in physical activities offered at work, suggesting that they have already established physical activity-related habits. Overweight and with obesity persons may require additional incentives and support to avail themselves of such opportunities [33]. Healthy snacks available in vending machines are more important for overweight and with obesity people. Similar findings were reported by Hanks et al. [36] who noted that stocking vending machines with healthier options resulted in a substantial increase in their selection by persons with higher BMI values. This suggests that interventions aimed at altering snack availability in the workplace could be an effective tool in promoting healthy eating habits among overweight and with obesity workers.

A look at barriers to healthy eating in the workplace revealed that, for overweight and with obesity people, the most significant barrier is a strained budget, which concurs with the findings reported by Cash et al. [37]. Their study showed that the higher cost of healthy food is a key factor limiting food choices among people with higher BMI values. These results suggest that workplace interventions such as subsidised healthy meals could be effective in reducing this barrier. Time constraints, which particularly affect with obesity individuals, have also been extensively studied. Sturm [38] found that a lack of time is one of the main barriers to sticking to healthy eating habits especially among overweight and with obesity people. Similar results were reported by Silva et al. [39]. The findings of the present study confirm that employers should consider strategies to reduce this barrier, such as offering healthy meals on site or providing the option to order healthy food. People with a normal BMI mention insufficient knowledge and motivation as the most important barriers. The study by Godin et al. [40] demonstrated that a lack of proper education and self-motivation to choose healthy eating can limit the effectiveness of health promotion programs. These results suggest that, for persons with a normal BMI, educational and motivational campaigns may be effective in maintaining healthy eating habits. Logistic constraints, such as the availability of healthy food, were more significant for individuals with a normal BMI. Similarly, Hilmers et al. [41] stressed the fact that the availability of healthy products is crucial for embracing healthy nutrition habits, especially among those who already have established dietary patterns.

## 5. Conclusions

Health promotion in the workplace is a combined effort of both employers and employees. Boosting employee health and well-being can be achieved through e.g., work organisation improvement, particularly in companies focused on achieving positive changes in employees’ health-related habits.

The study reported here revealed that larger enterprises are more inclined to establish meal consumption spaces, which contributes to a more organised work culture. The larger the business, the more time employees spend eating meals, it being influenced by better-developed eating facilities, which undoubtedly encourages the workers to take longer breaks. In such companies, the work is more varied, and responsibilities are distributed among a larger number of employees.

Overweight and with obesity people were found to place the highest value on healthy food options, which serves as a suggestion for companies to focus on creating such opportunities. People with a normal BMI are more responsive to healthy food subsidies, nutritional advice provided by a dietitian, and access to fitness facilities, indicating that financial and educational motivation is key. Workplaces should consider this when organising additional educational sessions to support these employees.

Understanding the differences between company sizes, and between various employee groups can assist in developing more effective and personalised strategies for promoting healthy eating in the workplace.

## 6. Limitations and Future Research

The present study is affected by certain limitations. Primarily, it pertains to Poland only, so the conclusions may not be applicable or extended to countries with higher or lower levels of development or different employee culture. Secondly, one has to bear in mind varied employee behaviours and trends in healthy lifestyles. Nutritional education research should be conducted systematically, and the dynamics of changes ought to be monitored as similar studies conducted in 10 years’ time may reveal entirely different behavioural patterns among employees and employers.

## Figures and Tables

**Table 1:** Characteristics of the research sample (data in %).

Gender	Employee’s BMI	Company			Total		
		Micro- and Small	Medium-Sized	Large	%	Mean	Standard Deviation
female	with obesity	9.09	4.45	6.44	19.98	6.660	2.328
	overweight	4.55	5.30	8.90	18.75	6.250	2.325
	normal	5.02	5.40	3.03	13.45	4.483	1.273
male	with obesity	9.09	5.02	6.91	21.02	7.007	2.037
	overweight	3.22	4.17	6.44	13.83	4.610	1.654
	normal	4.64	5.68	2.65	12.97	4.323	1.540
total		35.61	30.02	34.37	100.00		

Source: Own study based on the research.

**Table 2:** Type of support provided to employees by employers regarding healthy nutrition.

Specification	Wilks’ Lambda: 0.401 F = 20.490; *p* < 0.001 *			Company		
				Micro- and Small	Middle-Sized	Large
	Wilks’ Lambda	F Value	*p* Level	Classification Function		
meal break	0.471	45.787	0.001 *	2.978	4.169	4.777
complimentary food	0.406	3.463	0.032 *	0.148	0.583	0.445
training sessions	0.461	39.346	0.001 *	0.548	0.867	2.721
nutritionist	0.417	10.721	0.001 *	0.170	0.706	2.018
Constant				−1.861	−3.007	−3.620

*—level of significance of differences at *p* < 0.050. Source: Own study based on the research.

**Table 3:** Place for meal consumption according to the size of the company.

Company Size	Test Size N = 1056	Place for Meal Consumption at Work				
		I Do Not Eat Meals at Work	There Is a Special Space for Meal Consumption	At the Workstation During a Break	At the Workstation While Working	Other
		Chi-Square Test = 117.316; *p* < 0.001; V-Cramer’s = 0.236, Data in %				
micro- and small	376	18.35	15.43	32.71	31.12	2.39
middle-sized	317	7.89	30.28	45.11	14.51	2.21
large	363	4.68	28.37	52.62	11.02	3.31

Source: Own study based on the research.

**Table 4:** Time allocated to consume meals by employees.

Size of Company	Test Size N = 1056	I Do Not Eat Meals at Work	Up to 5 min	6–15 min	16–30 min	>30 min
		Chi-Square Test = 118.896; *p* < 0.001; Cramer’s V = 0.318, Data in %				
micro- and small	376	18.35	28.72	28.46	10.64	13.83
middle-sized	317	7.89	11.67	44.48	18.93	17.03
large	363	4.68	23.42	26.17	30.85	14.88

Source: Own study based on the research.

**Table 5:** Factors motivating employees to eat healthily in the workplace.

Factor	Wilks’ Lambda: 0.154 F = 324.16; *p* < 0.001 *			Employee’s BMI Indicating		
				The Person’s Weight Is Normal	The Person Is Overweight	The Person Is with Obesity
	Wilks’ Lambda	F Value	*p* Level	Classification Function		
availability of healthy food options in the company’s canteen/catering	0.223	232.816	<0.001	3.0250	5.950	6.2770
subsidised healthy food	0.271	96.541	<0.001	3.9130	2.241	2.7710
access to nutritionist counselling	0.183	93.588	<0.001	3.6090	1.917	2.3990
access to a fitness room	0.182	52.022	<0.001	3.1650	2.398	1.8750
healthy snacks in company vending machines/shop	0.215	205.692	<0.001	2.2460	3.613	4.9030
Constant				27.017	27.001	34.573

*—level of significance of differences at *p* < 0.050. Source: Own study based on the research.

**Table 6:** Barriers limiting healthy eating among employees in the workplace.

Barrier	Wilks’ Lambda: 0.136 F = 359.41; *p* < 0.001 *			Employee’s BMI Indicating		
				The Person’s Weight Is Normal	The Person Is Overweight	The Person Is with Obesity
	Wilks’ Lambda	F Value	*p* Level	Classification Function		
budget constraints	0.208	279.983	<0.001	2.403	5.700	5.954
time constraints	0.190	208.743	<0.001	2.449	3.851	5.121
lack of knowledge	0.172	139.055	<0.001	3.822	1.661	2.224
lack of motivation	0.165	111.793	<0.001	3.656	1.788	2.421
logistic limitations	0.151	58.361	<0.001	3.067	2.215	1.681
Constant				26.743	25.732	33.290

*—level of significance of differences at *p* < 0.050. Source: Own study based on the research.

## Data Availability

Data is available upon request.
